# Integrated Multi-Omics and Spatial Transcriptomics Identify FBLL1 as a Malignant Transformation Driver in Hepatocellular Carcinoma

**DOI:** 10.3390/cells15030246

**Published:** 2026-01-27

**Authors:** Junye Xie, Shujun Guo, Yujie Xiao, Yibo Zhang, An Hong, Xiaojia Chen

**Affiliations:** 1 Institute of Biomedicine & Department of Cell Biology, College of Life Science and Technology, Jinan University, Guangzhou 510632, China; xiejunyep@163.com (J.X.);; 2 State Key Laboratory of Bioactive Molecules and Druggability Assessment, Jinan University, Guangzhou 510632, China; 3 National Engineering Research Center of Genetic Medicine, Guangzhou 510632, China; 4 Guangdong Province Key Laboratory of Bioengineering Medicine, Guangzhou 510632, China; 5 Guangdong Provincial Biotechnology Drug & Engineering Technology Research Center, Guangzhou 510632, China; 6 MOE Key Laboratory of Tumor Molecular Biology, Jinan University, Guangzhou 510632, China

**Keywords:** hepatocellular carcinoma, FBLL1, spatial transcriptomics, malignant transformation, c-Myc, EGFR signaling

## Abstract

**Background**: Hepatocellular carcinoma (HCC) is characterized by marked intratumoral heterogeneity and poor clinical outcomes. Dysregulated ribosome biogenesis has emerged as a fundamental hallmark of tumor initiation and progression; however, the specific molecular drivers linking this machinery to HCC pathogenesis remain largely undefined. **Methods**: By integrating multi-omics data from the TCGA and ICGC cohorts, FBLL1 was identified as a key prognostic candidate gene. Its cellular and spatial distribution was analyzed using single-cell RNA sequencing and spatial transcriptomics. Its biological functions in vitro and in vivo were validated through functional experiments, including lentivirus-mediated ectopic expression and siRNA-mediated gene knockdown. Finally, its molecular mechanism was elucidated through transcriptomic analysis and Western blotting. **Results**: FBLL1 was significantly upregulated in HCC and correlated with poor patient survival. Spatial and single-cell analyses showed that FBLL1 expression was preferentially enriched in malignant hepatocytes within the tumor region. Functionally, knockdown FBLL1 could inhibit the proliferation and clonogenic capacity of HCC cells, while overexpression FBLL1 in non-tumorigenic hepatocytes could promote the tumorigenic phenotype in xenograft models. Transcriptomic analysis indicated that FBLL1 overexpression was associated with the synergistic upregulation of c-Myc and multiple EGFR ligands, as well as decreased expression of hepatocyte functional markers. Consistently, modulation of FBLL1 expression affected the activity of the EGFR–MAPK signaling pathway. **Conclusions**: Our study identifies FBLL1 as a previously unrecognized regulator associated with malignant state transition in HCC. Rather than acting as a direct regulator of core signaling components, FBLL1 is associated with ligand-dependent activation of the EGFR–MAPK pathway in conjunction with c-Myc upregulation. These findings indicate that FBLL1 represents a promising therapeutic target for disrupting oncogenic signaling programs in liver cancer.

## 1. Introduction

Hepatocellular carcinoma (HCC) is a major global health burden, ranking as the third leading cause of cancer-related mortality worldwide. In China, it remains the predominant cause of cancer-related mortality in males under the age of 60 [1]. Beyond its primary tumor burden, HCC is responsible for severe systemic complications, including cirrhosis-associated liver failure, portal hypertension, and profound cancer-induced cachexia, which collectively contribute to its dismal prognosis [1]. Due to the insidious onset and lack of early symptomatic indicators, over 70% of individuals are diagnosed with late-stage disease, at which point curative surgical interventions are no longer feasible options [2,3]. Although systemic therapies have improved in recent years, the 5-year survival rate remains under 20% [3]. Given the complex mutational landscape of HCC and the relative scarcity of druggable driver mutations, conventional mutation-based prognostic models often show limited reliability [4]. Consequently, identifying robust, non-mutational prognostic biomarkers and therapeutic targets is of critical importance.

Ribosomes are essential cellular machineries responsible for protein synthesis. Ribosome biogenesis involves multiple tightly regulated stages, including rRNA transcription, processing, and ribosome assembly, all of which are crucial for cell growth [5,6,7]. Increasing evidence has established dysregulated ribosome biogenesis as a key driver of oncogenesis [8,9,10]. For example, a cancer-specific form of Netrin-1 (ΔN-netrin-1) is found only in the nucleoli of cancer cells and supports malignant behavior [11]. The c-Myc oncogene enhances protein production by regulating ribosome biogenesis genes [12,13]. In addition, the rRNA methyltransferase Fibrillarin (FBL) has been linked to poor prognosis and aggressive behavior in multiple cancer types [14]. Although these findings underscore the importance of ribosome biogenesis in cancer development, ribosome biogenesis-associated regulators in HCC remain unclear. Notably, Fibrillarin-like 1 (FBLL1), a nucleolar protein and homolog of FBL, has not been systematically investigated in HCC or other solid tumors, yet its biological function and clinical relevance in cancer remain poorly defined.

In this study, we employed a multidimensional machine learning framework, integrating ten diverse algorithms into a comprehensive framework of 101 combinations to construct a robust ribosome-related gene signature (RRG) for HCC prognosis. By incorporating single-cell RNA sequencing, spatial transcriptomics, and proteomic data, we identified FBLL1, the homolog of FBL, as a key malignant driver enriched in the tumor niche. Functional experiments further demonstrated that FBLL1 promotes malignant transformation and hepatocyte dedifferentiation, potentially involving c-Myc/EGFR-associated signaling.

## 2. Materials and Methods

### 2.1. Cell Lines and Materials

The human hepatoma cell lines HepG2, SMMC-7721, Huh7, MHCC97H, MHCC97L were cultured in high-glucose Dulbecco’s modified Eagle medium (DMEM, Gibco, Waltham, MA, USA) supplemented with 10% fetal bovine serum (FBS, Gibco, Waltham, MA, USA) at 37 °C in a humidified atmosphere with 5% (*v*/*v*) CO_2_, while the human hepatic cell line MIHA was cultured under the same conditions in RPMI 1640 (Gibco) supplemented with 10% fetal bovine serum (FBS, Gibco). All cell lines were tested and confirmed free of mycoplasma.

### 2.2. Mouse HCC Xenografts

The animal study was conducted according to the ARRIVE (Animal Research: Reporting of In Vivo Experiments) guidelines and approved by the Ethics Committee of Animal Experiments of Jinan University, Guangzhou, China. (No. 20250523-06). MIHA-Vector/MIHA-FBLL1 cells (2 × 10^6^ cells/100 μL) were injected subcutaneously into 5-week-old male BALB/c nude mice (Guangdong Yaokang Biotechnology Co., Ltd., Guangzhou, China). The mice were euthanized on day 21.

### 2.3. Data Acquisition and Processing

Gene expression profiles and clinical annotation data for the TCGA-LIHC cohort were obtained from The Cancer Genome Atlas (TCGA, https://portal.gdc.cancer.gov/ (accessed on 5 May 2025)). External validation cohorts, including LIRI-JP and OEP000321, were obtained from the International Cancer Genome Consortium (ICGC, https://dcc.icgc.org/ (accessed on 5 May 2025)) and the Biomedical Data Sharing Platform (BMDC, https://www.biosino.org/bmdc/ (accessed on 5 May 2025)), respectively. To ensure comparability, RNA sequencing (RNA-seq) data were converted to transcripts per million (TPM) values. Differential expression analysis was performed using the “DESeq2” R package (v1.44.0), with a *p*-value < 0.05 and |log2Foldchange| ≥ 0.8 as the filtering criteria.

### 2.4. The Establishment of a Ribosome Biogenesis Gene Signature (RRG)

The following steps were used to establish a Ribosome-Related Gene Signature (RRGS). Firstly, the “survival” R package (v3.7.0) was utilized to conduct univariate Cox regression analysis, identifying differentially expressed genes (DEGs) with significant prognostic potential within the TCGA-LIHC cohort. Subsequently, the clinical samples were randomly partitioned into training and validation sets, ensuring a balanced distribution of survival and mortality events between the two groups.

The model was performed by using the “Mime” package (v0.12), which evaluated a comprehensive framework of 101 machine learning algorithm combinations. The performance of each candidate model was appraised via the C-index, and the optimal algorithm combination was selected based on the highest average C-index across trials. Following this, multivariate Cox regression analysis was applied to refine the gene selection and finalize the prognostic model. The individualized risk score for each patient was derived using the following weighted linear formula: risk score = 0.1781836 × FBLL1 + 0.2383363 × DDX11 + 0.4630241 × DCAF13.

We classified the HCC patients into high and low-risk groups based on the median risk score.

### 2.5. ScRNA Data Analysis

The GSE146115 and GSE166635 were downloaded from the GEO database (https://www.ncbi.nlm.nih.gov/geo/ (accessed on 5 May 2025)). The following steps were followed to analyze the scRNA data. The uniform manifold approximation and projection (UMAP) technique was used to visualize high-dimensional data in two-dimensional heatmaps. The uniform manifold approximation and projection (UMAP) technique was subsequently used to visualize the expression of the FBLL1, DCAF13 and DDX11 genes. The Kruskal-Wallis rank sum test was used to assess the differences in the expression of specific genes across different cell types. Finally, all cells were divided into positive and negative groups according to the expression of specific genes, and the proportion of each cell type in the positive and negative groups was calculated.

### 2.6. Spatial Transcriptomics Data Analysis

Spatial transcriptome analysis was performed via the Sparkle (https://grswsci.top/ (accessed on 10 May 2025)) and SpatialTME (https://www.spatialtme.yelab.site/ (accessed on 10 May 2025)). The processed gene expression data from the scRNA-seq and ST data were obtained from Mendeley Data (https://data.mendeley.com/datasets/skrx2fz79n/1 (accessed on 10 May 2025)). The analysis steps are as follows: Deconvolution analysis was utilized to accurately assess the cellular composition of each spot on the 10× Visium slides. We subsequently implemented strict quality control measures on the scRNA data on the basis of the number of expressed genes, UMI counts, and percentage of mitochondrial RNA. Next, we constructed a signature score matrix by calculating the average expression of the top 25 specific genes for each cell type in the scRNA-seq reference material across all spots. Finally, using the “Cottrazm” package, we generated an enrichment score matrix. The enrichment scores for each cell type were visualized via the spatial feature plot function from the Seu-rat package, where a higher enrichment score corresponded to a darker color, indicating a greater abundance of that cell type in the spot. Based on the expression score of each gene in the microregion, if the score for malignant cells in a microregion was 1, it was defined as the malignant group; if it was 0, it was defined as the normal group; otherwise, it was classified as the mixed group. We then employed Wilcoxon rank sum tests to assess the statistical significance of differences in the expression levels of specific genes between each pair of the three groups (malignant, normal, and mixed).

Based on the results of previous deconvolution, we calculated the cell type with the highest abundance in each microregion. For the expression level of each gene, we used the spatial feature plot function from the Seurat package and visualized the expression landscape of genes across each micro-region. Finally, we used Spearman correlation analysis and calculated the correlations between cellular abundances across all spots, as well as the correlations between cellular abundances and gene expression levels. For visualization, we utilized the “linkET” package.

### 2.7. Plasmid Transfection and RNA Interference

Stable cell line (MIHA-FBLL1) was established by lentiviral infection and selected with puromycin. For knockdown, siRNA were synthesized by Sangon Biotech (Shanghai, China) Co., Ltd. Transfections were performed using Lipofectamine™ 3000 (Invitrogen, Carlsbad, CA, USA) following the manufacturer’s protocol. The siRNA sequences are listed in Supplementary Table S1.

### 2.8. RNA Extraction and Quantitative Real-Time PCR

Total RNA was extracted using TRIzol (TaKaRa, Tokyo, Japan)). Reverse transcription was performed using the PrimeScript RT Reagent Kit (Takara, Tokyo, Japan). Relative expression levels were calculated using the 2^−ΔΔCt^ method. Primer sequences are provided in Supplementary Table S2.

### 2.9. Western Blotting

Total protein was extracted using RIPA lysis buffer containing protease and phosphatase inhibitors. Proteins were separated by SDS-PAGE and transferred onto PVDF membranes. The membranes were blocked with 5% BSA and incubated overnight at 4 °C with primary antibodies against Flag (1:2000, CST, #14793), c-Myc (1:1000, CST, #5605), Phospho-EGFR (Y1068) (1:1000, Abmart, T55232), EGFR (1:1000, Abmart, T55112), Phospho-ERK (1:1000, CST, #4377), ERK (1:1000, CST, #4695), β-actin (1:5000, Servicebio, GB-11001), and TUBB (1:5000, Servicebio, GB-11017). This was followed by incubation with HRP-conjugated secondary antibodies. Bands were visualized using an ECL detection system. Each experiment was repeated thrice independently. Raw data of Western blot are provided in Supplementary Figures S3–S5.

### 2.10. Cell Proliferation and Colony Formation Assays

For the CCK-8 assay, cells were seeded into 96-well plates. Cell viability was measured at 0, 24, 48, and 72 h using the Cell Counting Kit-8 (Dojindo, Kumamoto, Japan) by reading absorbance at 450 nm. For the colony formation assay, cells were seeded into 6-well plates (500 cells/well) containing DMEM (10% FBS). After 10 days, cells were fixed with paraformaldehyde and stained with crystal violet solution. Each experiment was repeated thrice independently.

### 2.11. Statistical Analysis

The data were analyzed via GraphPad Prism 8.0, and all the results are expressed as the means ± standard deviations. Differences between groups were measured via Student’s t test for comparisons or one-way ANOVA for multiple comparisons. The setting was not significantly different; *p* < 0.05 was considered statistically significant. * indicates *p* < 0.05, ** indicates *p* < 0.01 and *** indicates *p* < 0.001.

## 3. Results

### 3.1. Multi-Omics Profiling Identifies FBLL1 as a Prognostic Oncogenic Candidate in HCC

**Figure 1:**
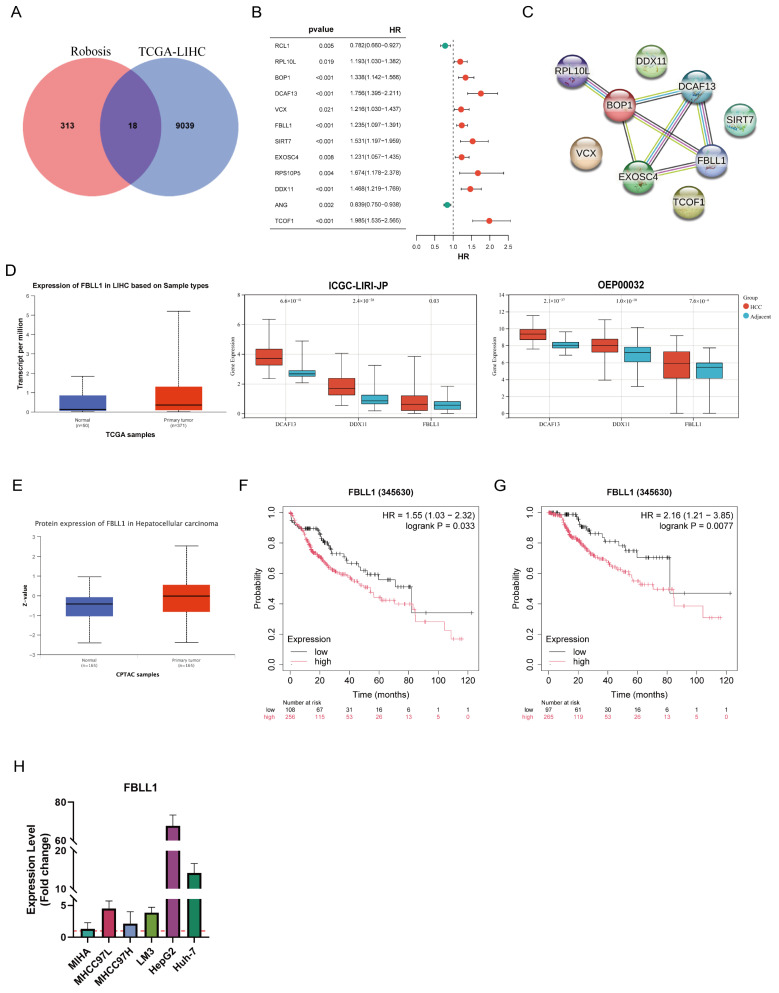
Multi-omics analysis identifies FBLL1 as a prognostic oncogene associated with ribosome biogenesis in HCC. (**A**) Venn diagram of overlapping genes between TCGA-LIHC DEGs and ribosome biogenesis gene sets. (**B**) Forest plot of univariate Cox regression for 12 candidate genes. (**C**) PPI network of candidate genes. (**D**) FBLL1 mRNA validation in ICGC and OEP cohorts. (**E**) FBLL1 protein abundance in the CPTAC cohort. (**F**,**G**) Kaplan–Meier analysis of overall survival (OS) and disease-specific survival (DSS) in TCGA-LIHC. (**H**) qPCR assay of FBLL1 in HCC cells vs. MIHA. Data are presented as mean ± SD.

To identify the key molecular drivers linking ribosome biogenesis to HCC progression, we performed a systematic multi-omics screening strategy. Differentially expressed genes (DEGs) from the TCGA-LIHC cohort were intersected with a curated ribosome biogenesis–related gene set, yielding 18 overlapping candidates (Figure 1A). Univariate Cox regression further narrowed them to 12 prognosis-associated candidates (Figure 1B), which formed a dense protein–protein interaction network (Figure 1C).

To identify the most critical prognostic factors, we implemented an integrative machine learning framework comprising 101 algorithmic combinations (Figure S1A). This approach generated a robust ribosome biogenesis–related gene signature (RRGs), among which the StepCox [both] + GBM model achieved strong prognostic performance using only four genes (RPL10L, FBLL1, DDX11, and DCAF13) (Figure S1B). Multivariate Cox regression analysis demonstrated that FBLL1, DDX11, and DCAF13 were independent prognostic factors for LIHC. Higher RRGs scores correlated with increased mortality and shorter survival (Figure S1C–E). To further validate the clinical relevance of the RRGs, we conducted comprehensive analyses incorporating clinicopathological features, including the construction of a nomogram based on multivariate Cox regression, univariate and multivariate Cox analyses, and receiver operating characteristic (ROC) curve analyses (Figures S2 and S6). While DCAF13 and DDX11 have been previously characterized in multiple cancers, the biological function of FBLL1 remains largely unexplored. Therefore, we focused on FBLL1 for in-depth investigation.

External datasets (ICGC-LIRI-JP, OEP000321 and CPTAC) confirmed that FBLL1 was significantly increased in tumor tissues compared to normal tissues (Figure 1D–F and Figure S7). Patients with high FBLL1 expression exhibited poorer overall survival (OS) and disease-specific survival (DSS). qRT-PCR assays further demonstrated that FBLL1 expression was significantly upregulated in HCC cells compared to MIHA (Figure 1H).

### 3.2. Single-Cell and Spatial Transcriptomics Reveal FBLL1 Enrichment in the Malignant Tumor Niches

**Figure 2:**
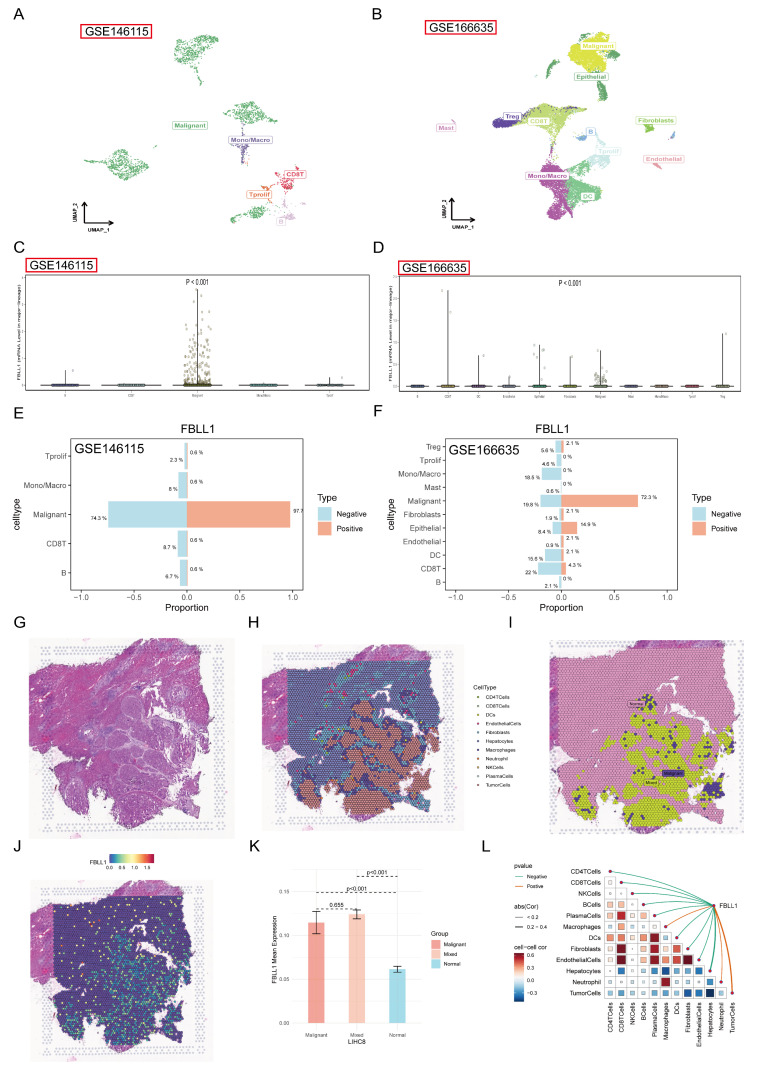
Spatial and cellular characterization of FBLL1 in the tumor microenvironment (**A**) UMAP visualization of cell clusters in HCC scRNA-seq data. (**B**) Violin plot of FBLL1 expression across cell types. (**C**) Spatial distribution of tissue clusters. (**D**) Spatial feature plot of FBLL1 expression. (**E**,**F**) Quantification of cell type proportions in FBLL1-positive vs. negative groups. (**G**–**I**) Spatial transcriptomics visualization showing tissue histology and cluster definitions. (**J**–**L**) Spatial feature plot of FBLL1 (**J**), with statistical comparisons between malignant and normal regions (**K**) and correlation analysis with tumor purity (**L**).

To elucidate the cellular distribution of FBLL1, we analyzed scRNA-seq and spatial transcriptomics. The scRNA-seq analysis clustered cells into major types, including malignant, immune, and stromal cells (Figure 2A,B). Notably, FBLL1 expression was highly specific in malignant cells, with low expression in non-malignant populations (Figure 2C,D). Stratifying cells into FBLL1-positive and -negative groups showed that malignant cells were markedly enriched in the positive fraction (Figure 2E,F).

Spatial transcriptomics further provided high-resolution positional information (Figure 2G–I). Spatial expression mapping visualization showed that FBLL1 was predominantly enriched in the malignant tumor niche (Figure 2J). Quantitative analyses showed that the average FBLL1 expression level was significantly higher in malignant regions than in adjacent normal regions, and FBLL1 expression was positively correlated with the proportion of tumor cells in each spatial spot (Figure 2K,L).

### 3.3. Knockdown of FBLL1 Inhibits HCC Proliferation and Clonogenicity In Vitro

**Figure 3:**
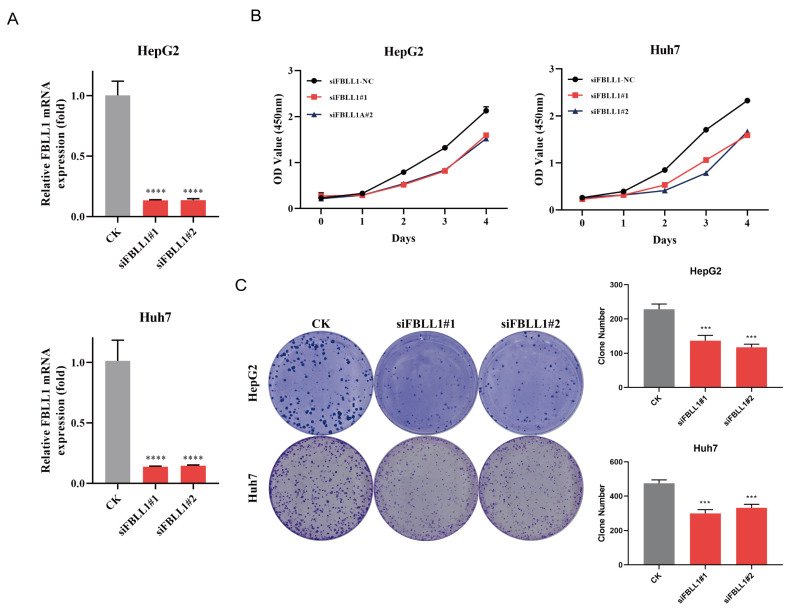
Knockdown of FBLL1 suppresses HCC cell proliferation (**A**) Verification of FBLL1 knockdown efficiency. (**B**) CCK-8 proliferation assay. (**C**) Colony formation assay. Data are presented as mean ± SD of three independent experiments. *** *p* < 0.001, **** *p* < 0.0001.

To investigate the functional requirement of FBLL1 in HCC, siRNA-mediated knockdown was performed in HepG2 and Huh7 cells. Owing to the absence of commercially available antibodies for detecting endogenous FBLL1, silencing efficiency was validated at the mRNA level by qRT-PCR (Figure 3A).

Functionally, Loss of FBLL1 significantly inhibited cell proliferation in a time-dependent manner (Figure 3B) and drastically reduced colony-forming ability (Figure 3C). These results indicate that FBLL1 is crucial for maintaining the growth of HCC cells.

### 3.4. FBLL1 Drives Malignant Transformation and Tumorigenesis In Vivo

**Figure 4:**
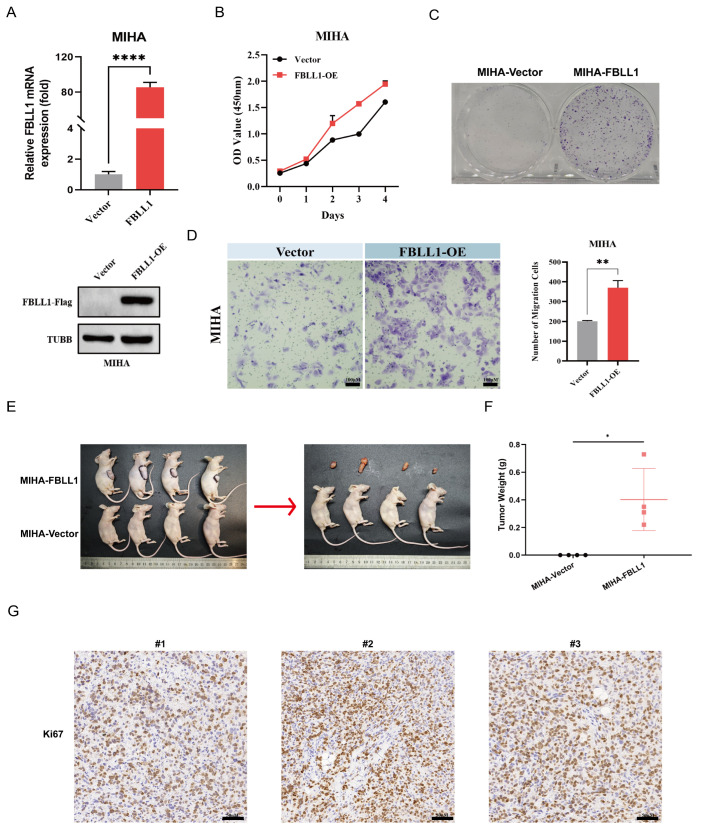
FBLL1 promotes malignant transformation and tumorigenesis (**A**) Western blot validation of Flag-FBLL1 expression in MIHA cells using an anti-Flag antibody. (**B**) CCK-8 proliferation assay. (**C**) Colony formation assay. (**D**) Transwell migration assay. (**E**) Representative images of subcutaneous xenograft tumors (*n* = 4/group). (**F**) Quantification of tumor weights. (**G**) Representative Ki67 IHC staining of tumor tissues. Data are presented as mean ± SD, * *p* < 0.05, ** *p* < 0.01, **** *p* < 0.0001.

To determine whether FBLL1 is sufficient to drive tumorigenesis, we constructed a stable MIHA cell line which overexpressing FBLL1 (Figure 4A). In vitro, FBLL1 overexpression significantly enhanced cell proliferation (Figure 4B), colony formation ability (Figure 4C) and migration ability (Figure 4D) of MIHA cells. Furthermore, MIHA-Vector and MIHA-FBLL1 cells were subcutaneously injected into nude mice. While MIHA cells failed to form tumors, FBLL1-overexpressing group generated palpable xenograft tumors (Figure 4E,F). Immunohistochemical staining for Ki67 revealed a high proliferative index in the FBLL1-driven tumors (Figure 4G).

### 3.5. FBLL1 Overexpression Is Associated with c-Myc Upregulation and EGFR–MAPK Activation

**Figure 5:**
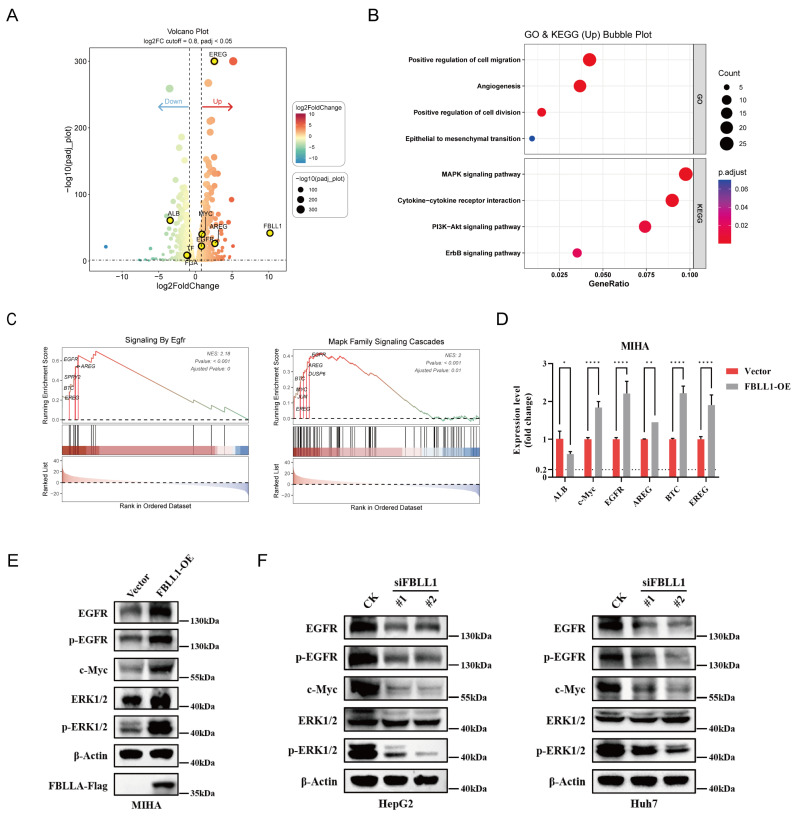
FBLL1 activates the c-Myc/EGFR signaling axis (**A**) Volcano plot of DEGs (MIHA-Vector vs. MIHA-FBLL1). (**B**) GO and KEGG analyses of upregulated genes. (**C**) GSEA of KRAS, EGFR and MAPK signaling. (**D**) qPCR validation of key targets in MIHA cells. (**E**,**F**) Western blot of c-Myc, p-EGFR, and p-ERK in FBLL1-overexpressing and knockdown cells. Data are presented as mean ± SD. * *p* < 0.05, ** *p* < 0.01, **** *p* < 0.0001.

To characterize the changes associated with FBLL1 overexpression, we performed RNA sequencing on MIHA-Vector and MIHA-FBLL1 cells. Differential expression analysis revealed extensive transcriptional reprogramming in response to FBLL1 overexpression (Figure 5A). Among the downregulated genes, the hepatocyte differentiation marker ALB showed a marked decrease (log_2_FC ≈ −3.45), suggesting a potential shift away from a mature hepatocyte transcriptional profile.

Concomitantly, we observed increased expression of components associated with the c-Myc and EGFR signaling axis. MYC transcripts were upregulated (log_2_FC ≈ 0.90), as well as EGFR (log_2_FC ≈ 0.82) and its ligands, including AREG (log_2_FC ≈ 2.66), EREG (log_2_FC ≈ 2.58), and BTC (log_2_FC ≈ 1.63). Functional enrichment analyses (GO and KEGG) indicated that upregulated genes were significantly enriched in pathways related to the positive regulation of cell division and MAPK signaling (Figure 5B). Consistently, gene set enrichment analysis (GSEA) revealed significant enrichment of KRAS and EGFR signaling signatures in FBLL1-overexpressing cells (Figure 5C).

qRT–PCR was used to confirm the altered expression of MYC, EGFR, AREG, EREG, and ALB (Figure 5D). Also, c-Myc abundance and increased phosphorylation of EGFR and ERK1/2 in FBLL1-overexpressing cells at the protein level, (Figure 5E). Conversely, FBLL1 knockdown in HCC cells will reduce c-Myc protein levels and suppress of EGFR and ERK1/2 phosphorylation (Figure 5F), supporting a consistent association between FBLL1 expression and EGFR–MAPK pathway activity.

## 4. Discussion

Accumulating evidence has linked the dysregulation of ribosome biogenesis to the development of cancer [8,9,10,15]. Beyond supporting elevated biosynthetic demand, ribosome-associated factors are increasingly recognized as active regulators of oncogenic cell states through selective translational control and RNA modification. However, the exact mechanisms by which ribosome-related regulators contributes to malignant progression are still unclear [14]. In this study, we integrated multi-omics data with machine learning techniques to create and validate a four-gene prognostic signature, referred to as RRGs (RPL10L, FBLL1, DDX11, and DCAF13). RRGs effectively categorize HCC patients into different risk groups with significantly different prognoses. Additionally, single-cell and spatial transcriptomic analyses confirmed that FBLL1 is specifically enriched in the malignant niches, suggesting a localized role in tumor maintenance. This spatial specificity suggests that FBLL1 may play an environment-dependent role in maintaining the malignant cell state.

Among the genes associated with RRGs, RPL10L has received limited attention due to low expression [16]. In contrast, DCAF13 [17,18,19,20] and DDX11 [21,22,23,24] have been extensively studied as facilitators of tumor progression. However, the involvement of FBLL1 in cancer has not been previously documented. RNA 2′-O-methylation (Nm-modification) plays a crucial role as a posttranscriptional modification affecting cell fate [25,26,27]. In mammals, this is catalyzed by enzymes like FBL and FBLL1 within the Box C/D snoRNP complex [27,28,29]. Structural studies indicate that, compared with FBL, FBLL1 may have distinct substrate specificity and biological functions [29]. While the roles of FBL in cancer are well documented [14,30,31], FBLL1 had not been systematically studied in HCC or other solid tumors previously. Emerging evidence indicates that aberrant Nm modification can selectively enhance the translation of oncogenic transcripts, particularly those harboring internal ribosome entry site (IRES) elements, such as *MYC* [26,32]. Our findings support this emerging model of selective translational regulation. Specifically, FBLL1 overexpression led to a significant increase in c-Myc protein abundance, while changes in mRNA levels were relatively small, consistent with translational rather than transcriptional regulation. This observation aligns with recent evidence indicating that FBLL1 directly binds to the IRES of c-Myc, promoting IRES-dependent translation, thus positioning FBLL1 as an upstream regulator of oncogenic translational programs, rather than a passive component of protein synthesis.

Interestingly, we found that FBLL1 overexpression induced a coordinated upregulation of multiple EGFR ligands, including AREG, EREG and BTC, which creates a strong loop that keeps the EGFR pathway active. Furthermore, we found significant downregulation of ALB (log_2_FC ≈ −3.45),Transferrin (TF, log_2_FC ≈ −1.16) and Fibrinogen Alpha Chain (FGA, log_2_FC ≈ −1.06). These results indicate a shift in the transcriptional profile of hepatocytes. This transcriptional reprogramming is consistent with a transition of the cells towards a more progenitor-like or plastic state.

However, limitations must be acknowledged. Due to the lack of high-quality endogenous antibodies, FBLL1 knockdown efficiency was validated at the mRNA level. Moreover, the precise RNA substrates undergoing FBLL1-mediated Nm modification in HCC remain to be identified. Future studies employing direct RNA modification mapping and pharmacological dependency assays will be necessary to further delineate the causal hierarchy between FBLL1 activity, selective translation, and downstream oncogenic signaling pathway.

## 5. Conclusions

This study integrates multi-omics analysis, single-cell and spatial transcriptomics, and functional experiments to reveal that FBLL1 is a previously underestimated oncogenic factor in hepatocellular carcinoma (HCC). By combining bulk transcriptome analysis with spatial and cellular resolution, we found that FBLL1 is preferentially enriched in the malignant tumor microenvironment and is closely associated with aggressive tumor phenotypes. Functional experiments further confirmed that FBLL1 plays an active role in the development and progression of liver cancer. Overexpression of FBLL1 in hepatocytes is sufficient to induce malignant transformation and tumor formation in vivo, while knockdown of FBLL1 significantly inhibits the proliferation and colony formation ability of liver cancer cells in vitro. These findings suggest that FBLL1 may be a functional driver of liver cancer. Mechanistically, RNA-Seq showed that FBLL1 promotes a c-Myc/EGFR-related transcriptional program, characterized by the upregulation of c-Myc, EGFR, and multiple EGFR ligands (including AREG and EREG), while simultaneously suppressing the expression of hepatocyte differentiation markers. Consistent with this, modulating FBLL1 expression directly affects the activation of the EGFR-MAPK pathway, highlighting the functional link between FBLL1 and growth factor signaling in liver cancer. In summary, our study establishes a previously unrecognized link between a ribosome biogenesis-related factor and the oncogenic signaling network in hepatocellular carcinoma. This work not only provides mechanistic insights into FBLL1-mediated tumor progression but also suggests that FBLL1 may serve as a potential prognostic indicator and therapeutic target for liver cancer.

## Data Availability

The original data presented in the study are openly available in SRA database at PRJNA1397508.

## Supplementary Materials

The following supporting information can be downloaded at: https://www.mdpi.com/article/10.3390/cells15030246/s1, Figure S1: Construction of HCC RRG by developing a competitive machine learning framework; Figure S2: Analysis of the correlation between the RRG prognostic model and clinical features; Figure S3. Raw data of western blot related to Figure 4A; Figure S4. Raw data of western blot related to Figure 5E; Figure S5. Raw data of western blot related to Figure 5F; Figure S6. Clinical Correlation of FBLL1 in HCC; Figure S7. Pan-cancer expression landscape of FBLL1; Table S1: qPCR Primer; Table S2: siRNA-Sequence.
