# Supplementary material for: Integrated Multi-Omics and Spatial Transcriptomics Identify FBLL1 as a Malignant Transformation Driver in Hepatocellular Carcinoma
Source: Cells. 2026 Jan 27;15(3):246. doi: 10.3390/cells15030246 (PMC12897225; doi:10.3390/cells15030246)
Supplement: Supplementary file 1 [file cells-15-00246-s001.zip › cells-4100459-supplementary.pdf]

Supplemental Information

Integrated Multi-omics and Spatial Transcriptomics Reveal FBLL1 Drives Malignant Transformation in Hepatocellular Carcinoma via the c-Myc/EGFR Axis

Junye Xie[1-6#], Shujun Guo[1-6#], Yujie Xiao[1-6], Yibo Zhang[1-6], An Hong[1-6*], Xiaojia Chen[1-6*]

[1] Institute of Biomedicine & Department of Cell Biology, College of Life Science and Technology, Jinan University, Guangzhou 510632, China

[2] State Key Laboratory of Bioactive Molecules and Druggability Assessment, Jinan University, Guangzhou 510632, China

[3] National Engineering Research Center of Genetic Medicine, Guangzhou 510632, China

[4] Guangdong Province Key Laboratory of Bioengineering Medicine, Guangzhou 510632, China

[5] Guangdong Provincial Biotechnology Drug & Engineering Technology Research Center, Guangzhou 510632, China

[6] MOE Key Laboratory of Tumor Molecular Biology, Jinan University, Guangzhou 510632, China

[#] These authors contributed equally to this work.

[*] **Corresponding author:** tha@jnu.edu.cn (A.H.); tchenxj@jnu.edu.cn (X.C.)

Supplementary Figures

Supplementary Figure S1. Construction of HCC RRG by developing a competitive machine learning framework

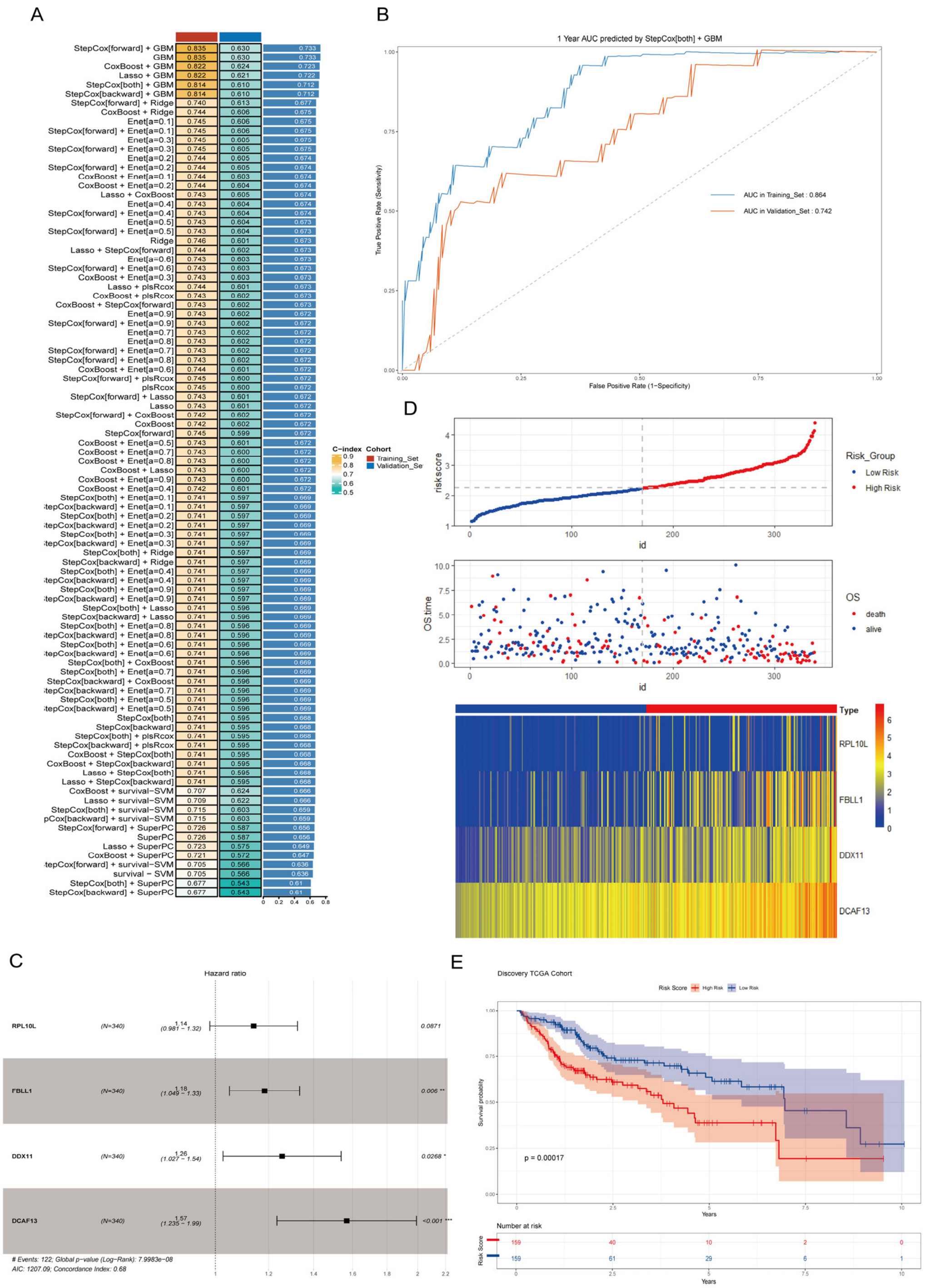

(A) A total of 101 kinds of prediction signatures were identified via a tenfold cross-

validation framework, and the C-index of each signature across the validation datasets was further calculated. (B) ROC curve of the StepCox[both] + GBM model features. (C) Multivariate regression analysis of the StepCox[both] + GBM model. (D) Risk factor plot of the TCGA-LIHC cohort. (E) Kaplan–Meier curves of OS according to the RRGS in the TCGA-LIHC cohort.

Supplementary Figure S2. Analysis of the correlation between the RRG prognostic model and clinical features

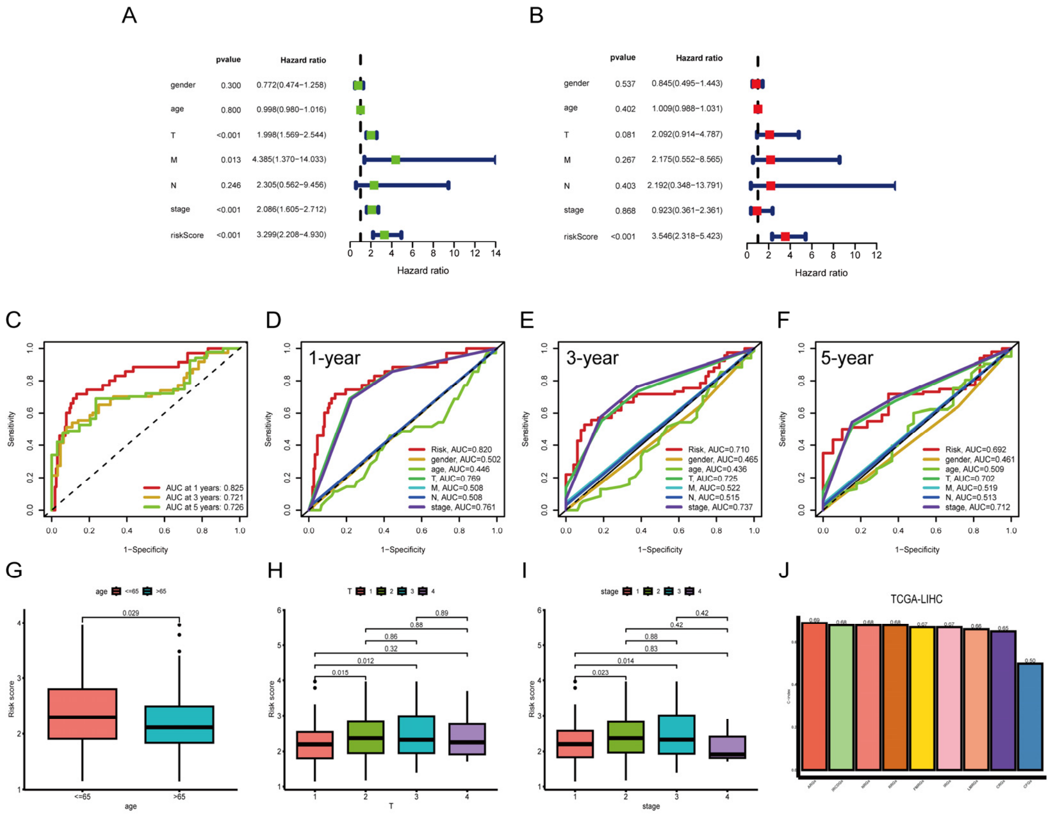

(A) Univariate Cox analysis of RRGs. (B) Cox multivariate analysis of RRGs. (C-F) ROC curves showing the specificity and sensitivity of the RRGS and clinical characteristics in predicting 1-, 2-, and 3-year OS in the TCGA-LIHC cohort. (G-I) Correlation analysis of age, overall cancer stage, T stage and risk score. (J) C-index of 9 prognostic signatures in the TCGA-LIHC cohort.

Supplementary Figure S3. Raw data of western blot related to Figure. 4A

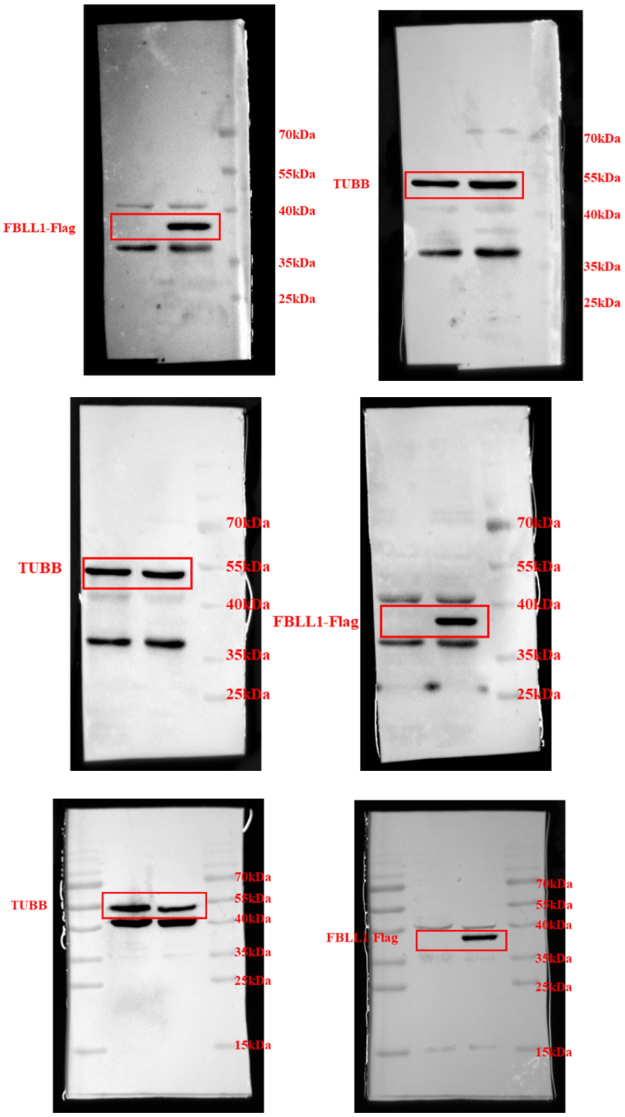

Supplementary Figure S4. Raw data of western blot related to Figure. 5E

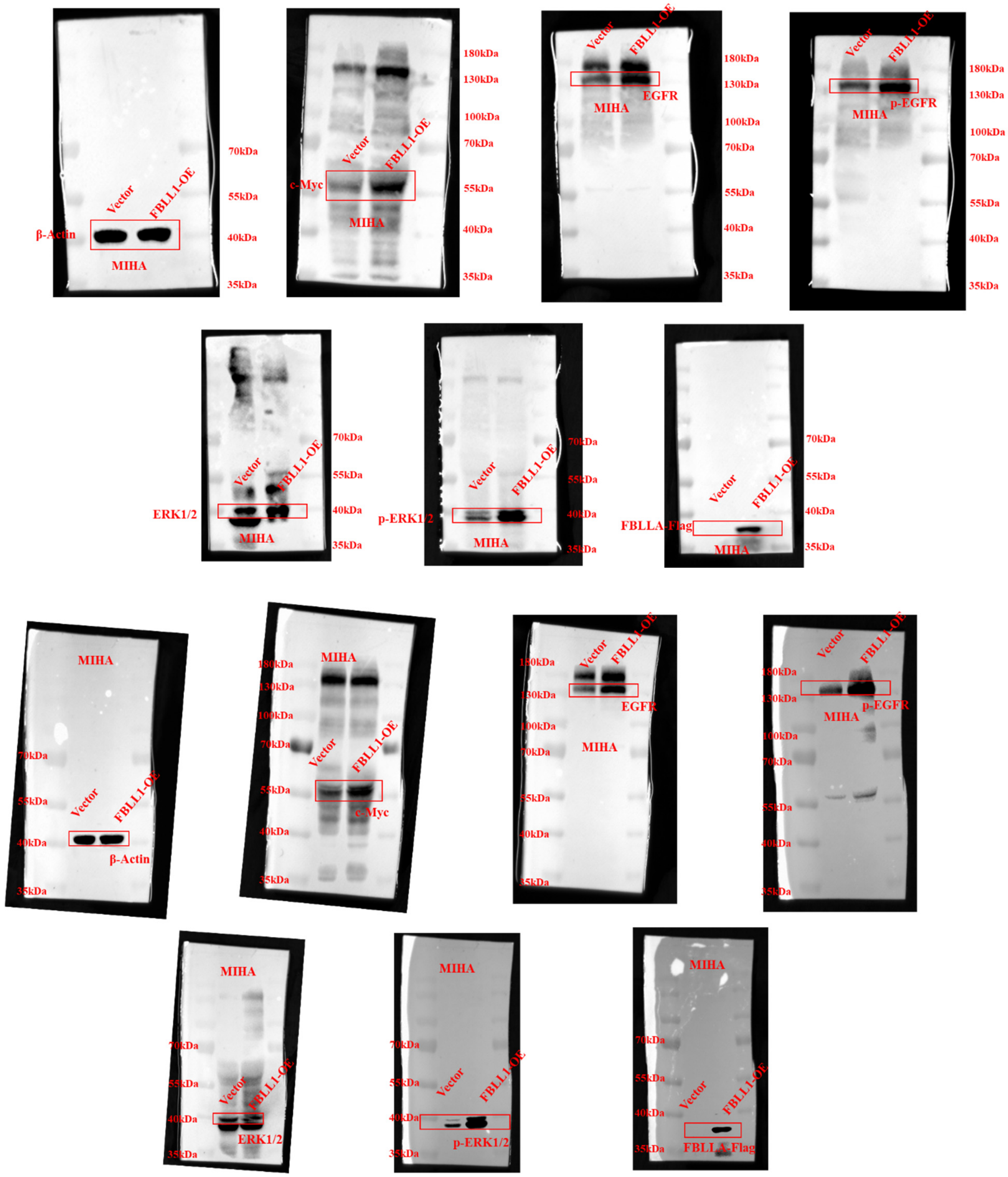

Vector
FBLL1-OE
β-Actin
MIHA
70kDa
55kDa
40kDa
35kDa
Vector
FBLL1-OE
c-Myc
MIHA
180kDa
130kDa
100kDa
70kDa
55kDa
40kDa
35kDa
Vector
FBLL1-OE
EGFR
MIHA
180kDa
130kDa
100kDa
70kDa
55kDa
40kDa
35kDa
Vector
FBLL1-OE
p-EGFR
MIHA
180kDa
130kDa
100kDa
70kDa
55kDa
40kDa
35kDa
Vector
FBLL1-OE
ERK1/2
MIHA
70kDa
55kDa
40kDa
35kDa
Vector
FBLL1-OE
p-ERK1/2
MIHA
70kDa
55kDa
40kDa
35kDa
Vector
FBLL1-OE
FBLLA-Flag
MIHA
70kDa
55kDa
40kDa
35kDa
MIHA
Vector
FBLL1-OE
β-Actin
70kDa
55kDa
40kDa
35kDa
MIHA
Vector
FBLL1-OE
c-Myc
180kDa
130kDa
100kDa
70kDa
55kDa
40kDa
35kDa
Vector
FBLL1-OE
EGFR
MIHA
180kDa
130kDa
100kDa
70kDa
55kDa
40kDa
35kDa
Vector
FBLL1-OE
p-EGFR
MIHA
180kDa
130kDa
100kDa
70kDa
55kDa
40kDa
35kDa
MIHA
Vector
FBLL1-OE
ERK1/2
70kDa
55kDa
40kDa
35kDa
MIHA
Vector
FBLL1-OE
p-ERK1/2
70kDa
55kDa
40kDa
35kDa
MIHA
Vector
FBLL1-OE
FBLLA-Flag
70kDa
55kDa
40kDa
35kDa

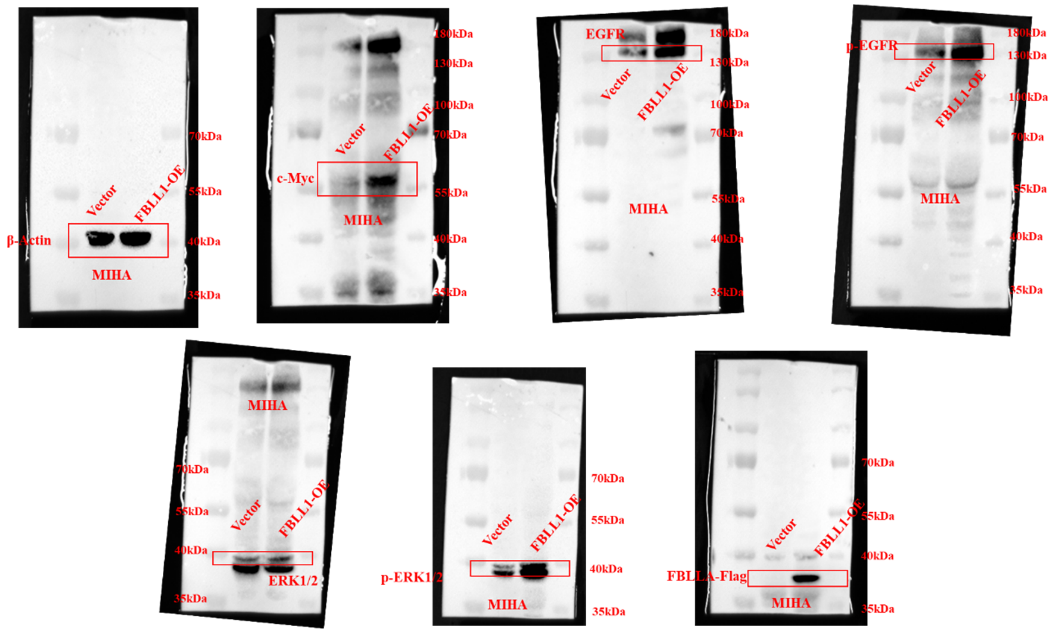

Supplementary Figure S5. Raw data of western blot related to Figure. 5F

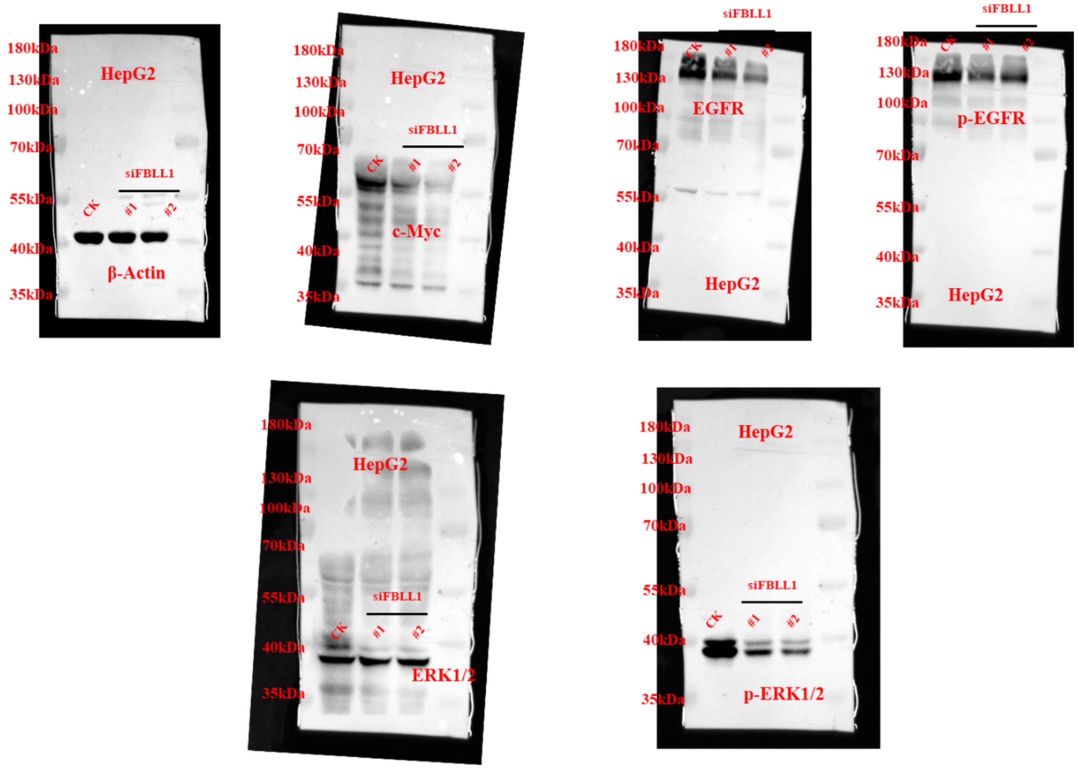

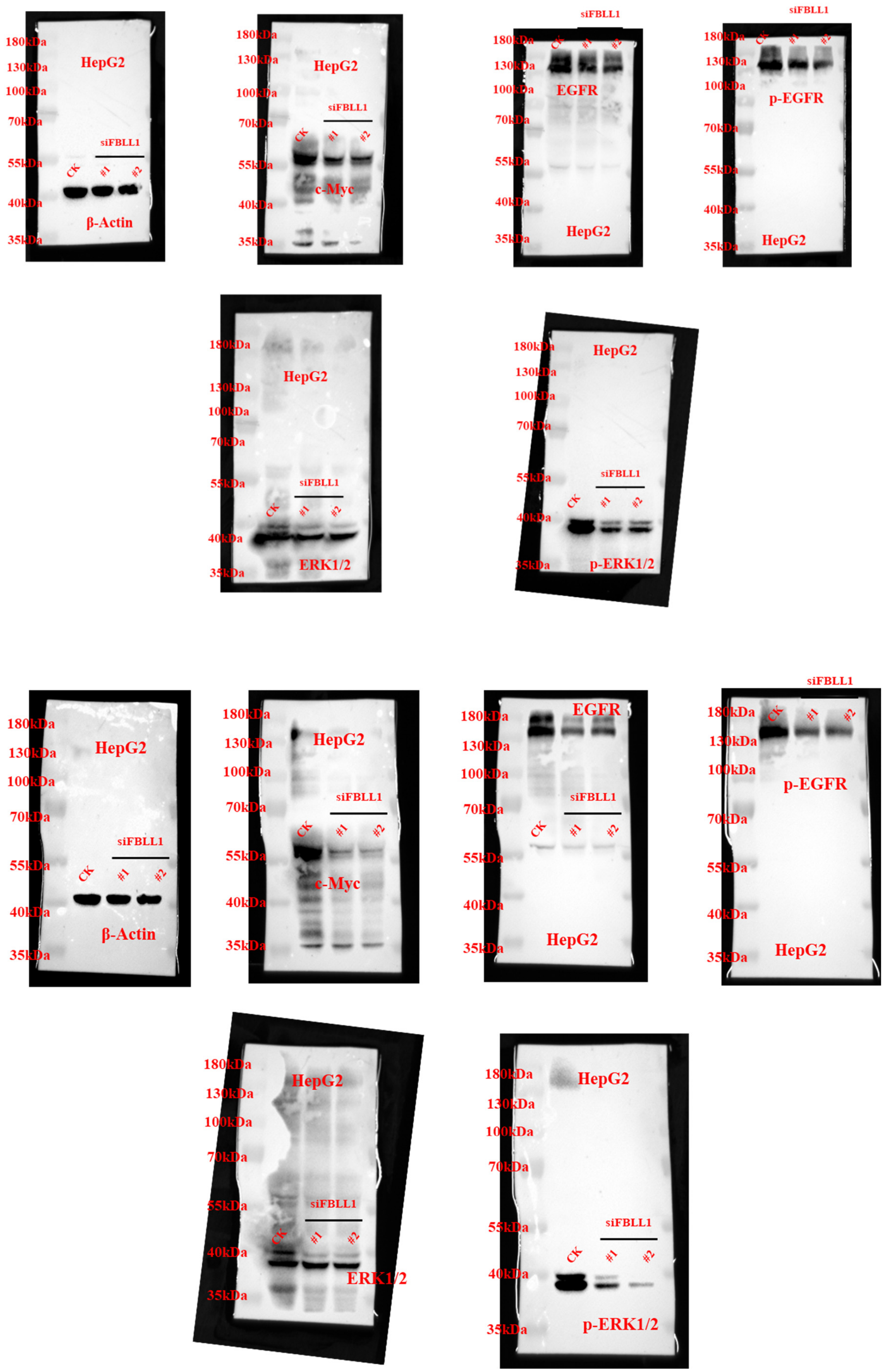
HepG2 β-Actin siFBLL1 CK #1 #2 180kDa 130kDa 100kDa 70kDa 55kDa 40kDa 35kDa
HepG2 c-Myc siFBLL1 CK #1 #2 180kDa 130kDa 100kDa 70kDa 55kDa 40kDa 35kDa
HepG2 EGFR siFBLL1 CK #1 #2 180kDa 130kDa 100kDa 70kDa 55kDa 40kDa 35kDa
HepG2 p-EGFR siFBLL1 CK #1 #2 180kDa 130kDa 100kDa 70kDa 55kDa 40kDa 35kDa
HepG2 ERK1/2 siFBLL1 CK #1 #2 180kDa 130kDa 100kDa 70kDa 55kDa 40kDa 35kDa
HepG2 p-ERK1/2 siFBLL1 CK #1 #2 180kDa 130kDa 100kDa 70kDa 55kDa 40kDa 35kDa
HepG2 β-Actin siFBLL1 CK #1 #2 180kDa 130kDa 100kDa 70kDa 55kDa 40kDa 35kDa
HepG2 c-Myc siFBLL1 CK #1 #2 180kDa 130kDa 100kDa 70kDa 55kDa 40kDa 35kDa
HepG2 EGFR siFBLL1 CK #1 #2 180kDa 130kDa 100kDa 70kDa 55kDa 40kDa 35kDa
HepG2 p-EGFR siFBLL1 CK #1 #2 180kDa 130kDa 100kDa 70kDa 55kDa 40kDa 35kDa
HepG2 ERK1/2 siFBLL1 CK #1 #2 180kDa 130kDa 100kDa 70kDa 55kDa 40kDa 35kDa
HepG2 p-ERK1/2 siFBLL1 CK #1 #2 180kDa 130kDa 100kDa 70kDa 55kDa 40kDa 35kDa

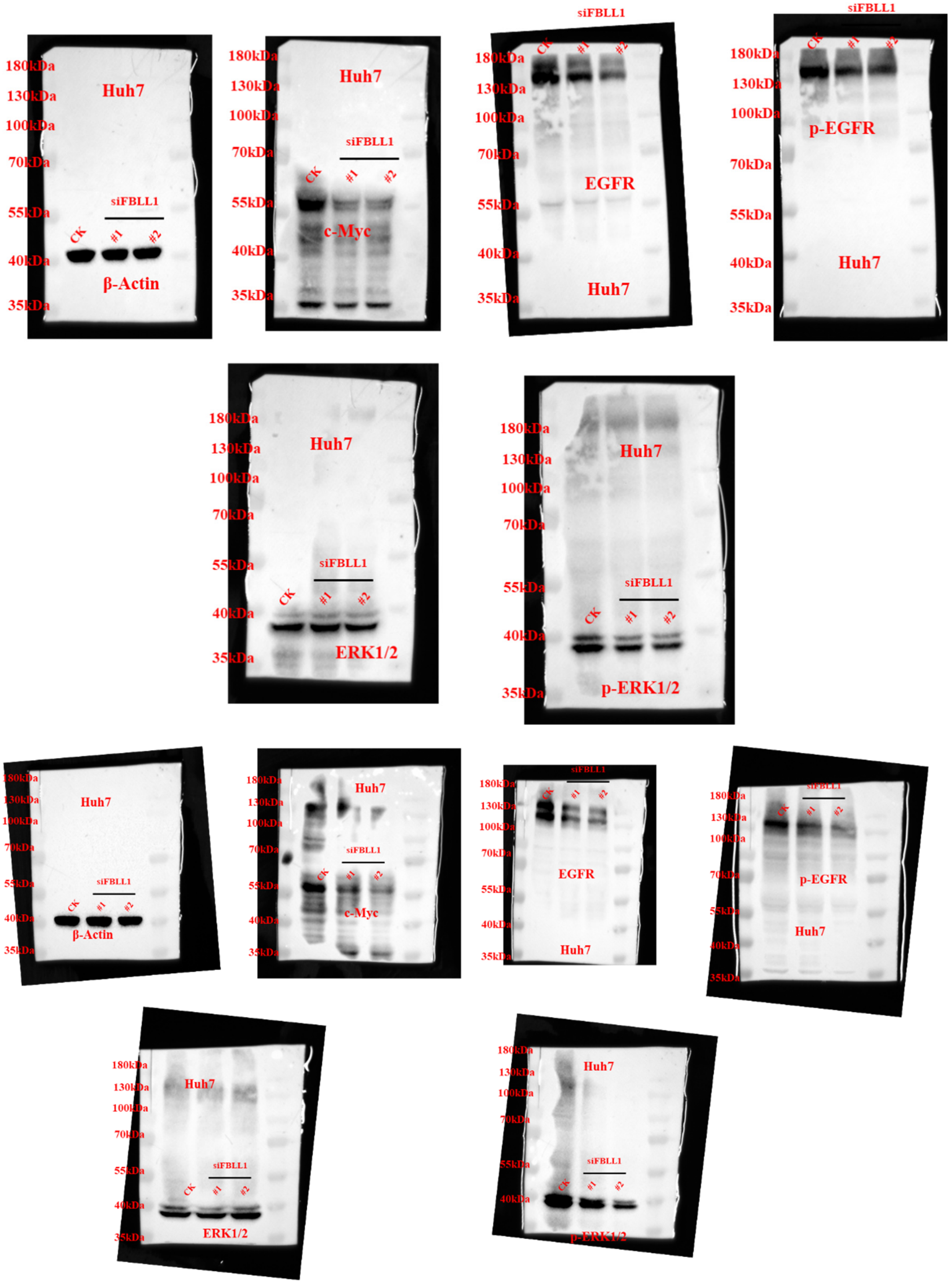
Huh7
siFBLL1
CK
#1
#2
β-Actin
c-Myc
EGFR
p-EGFR
ERK1/2
p-ERK1/2
180kDa
130kDa
100kDa
70kDa
55kDa
40kDa
35kDa

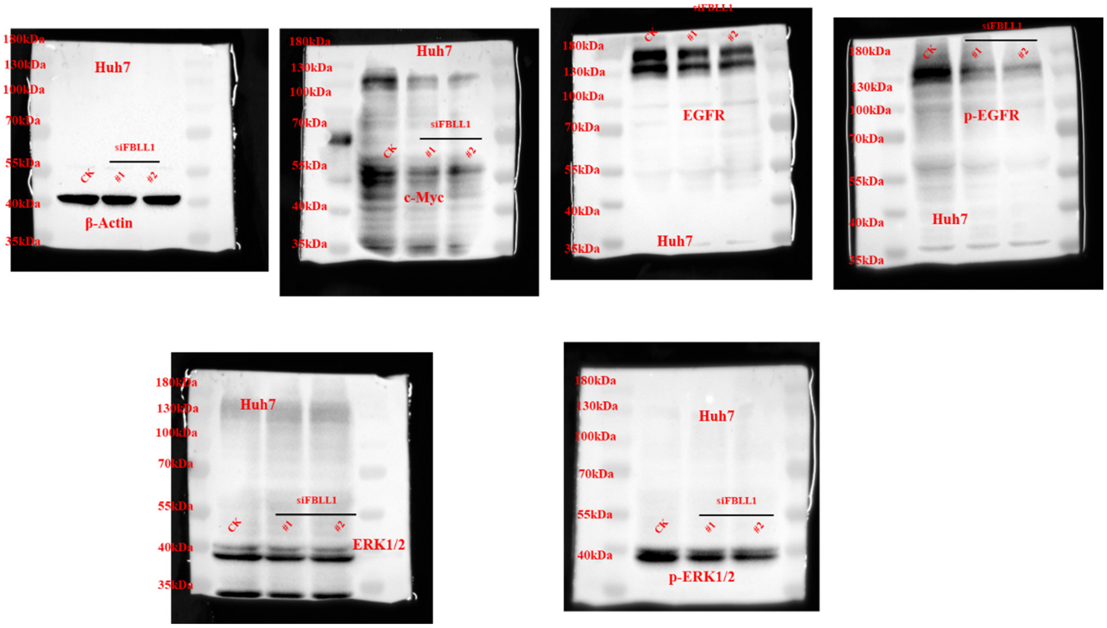

Supplementary Figure S6. Clinical Correlation of FBLL1 in HCC

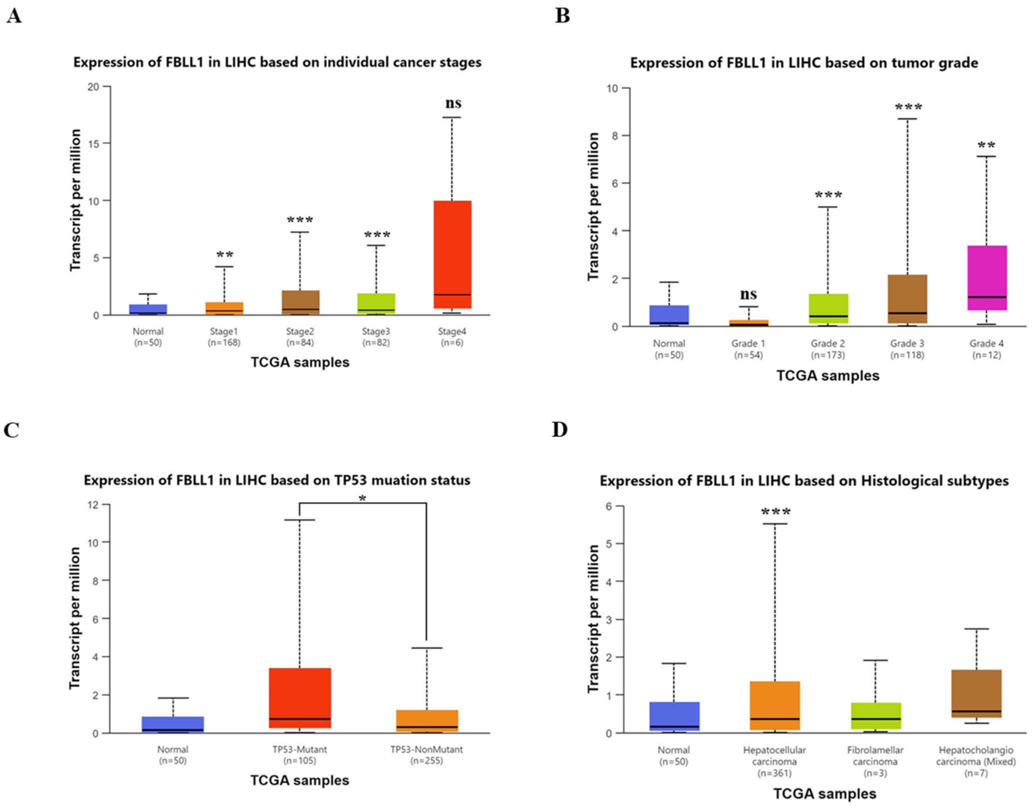

(A) Expression of FBLL1 across individual cancer stages. (B) FBLL1 expression correlates with tumor grade. (C) TP53 mutation status. (D) Distribution of FBLL1

across histological subtypes.

Supplementary Figure S7. Pan-cancer expression landscape of FBLL1

A

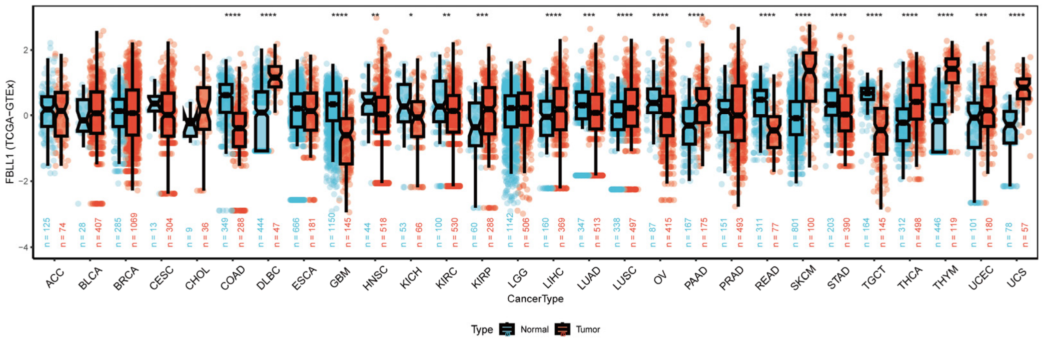

(A) Differential mRNA expression profile of FBLL1 across various cancer types and corresponding normal tissues from the TCGA and GTEx datasets. Normal samples are indicated in blue, and tumor samples are indicated in red. Statistical significance was assessed using the Wilcoxon test (**p<0.05, **p<0.01, ***p<0.001, ****p<0.0001*).

Supplementary Table S1: qPCR Primer

**qPCR Primer**	**Sequence (5' to 3')**
Actin-F	AAGGATTCCTATGTGGGCGAC
Actin-R	CGTACAGGGATAGCACAGCC
MYC-F	CAAGAGGCGAACACACAACG
MYC-R	CAACTCCGGGATCTGGTCAC
EGFR-F	CGAGTACCTCATCCCACAGC
EGFR-R	CACGGTGGAATTGTTGCTGG
AREG-F	TCCACTCGCTCTTCCAACAC
AREG-R	GGGGCTCTCATTGGTCCTTC
EREG-F	CACAGTCGTCGGTTCCACAT
EREG-R	TTCAGACTTGCGGCAACTCT
BTC-F	TGGTGGCAGATGGGAATTCC
BTC-R	CCGCTTTGATTGTGTGGTGG
ALB-F	TCTTCTGTCAACCCCACACG
ALB-R	ATCTCGACGAAACACACCCC

Supplementary Table S2 :siRNA- Sequence

Primer	Sequence
FBLL1-s#1	GCGUUGUGUUUGCUAUUAUUU
FBLL1-a#1	AUAAUAGCAAACACAACGCAG
FBLL1-s#2	GGCUGCGUUGUGUUUGCUAUU
FBLL1-a#2	UAGCAAACACAACGCAGCCUU
